# Buckley-James Estimator of AFT Models with Auxiliary Covariates

**DOI:** 10.1371/journal.pone.0104817

**Published:** 2014-08-15

**Authors:** Kevin Granville, Zhaozhi Fan

**Affiliations:** 1 Department of Statistics and Actuarial Science, University of Waterloo, Waterloo, Ontario, Canada; 2 Department of Mathematics and Statistics, Memorial University, St. John's, Newfoundland, Canada; Cleveland Clinic Lerner Research Institute, United States of America

## Abstract

In this paper we study the Buckley-James estimator of accelerated failure time models with auxiliary covariates. Instead of postulating distributional assumptions on the auxiliary covariates, we use a local polynomial approximation method to accommodate them into the Buckley-James estimating equations. The regression parameters are obtained iteratively by minimizing a consecutive distance of the estimates. Asymptotic properties of the proposed estimator are investigated. Simulation studies show that the efficiency gain of using auxiliary information is remarkable when compared to just using the validation sample. The method is applied to the PBC data from the Mayo Clinic trial in primary biliary cirrhosis as an illustration.

## Introduction

It is not uncommon to have one or more missing or mismeasured covariates in large cohort epidemiological studies. There are always cases in medical studies, where it is difficult to obtain an accurate measurement for all patients due to a procedure being too expensive or invasive. Alternatively, some auxiliary measurements which are less precise, but highly related to the target procedure, can be easily collected. In some situations, all of the measurements are error prone, while in other cases, a validation subsample, where the measurements are all accurately taken, is made available.

The former is a pure measurement error problem. The purpose of this paper is to investigate the inference of the latter cases in a failure time setting. In some cases, the validation sample could be large enough on its own, so one could choose to ignore all data from subjects that have missing or mismeasured values for any of the covariates, with just a minor efficiency loss. However, if the validation sample is relatively small, utilizing the auxiliary information will lead to remarkable efficiency gain, as our simulation results will show.

The literature on statistical inference of missing or mismeasured data of failure time regression models is abundant. Ignoring the measurement errors in modeling could lead to severe estimation bias, depending on the magnitude of the measurement error, hence invalidate the whole inference procedure ([1] Prentice, 1982). See also [2] (Rubin, 1976), [3] (Fuller, 1987), [4] (Carrol et al., 1995), and [5] (Wang et al., 1998) among others. The negative influence of mismeasured or missing covariates is largely understood for the Cox proportional hazards model. But the same cannot be said of accelerated failure time models. Details about the Cox model can be seen in the work of [6] (Cox, 1972), [7] (Cox and Oakes, 1984), [8] (Kalbfleisch and Prentice, 2002), [9] (Hu et al., 1998), and [10] (Hu and Lin, 2002), and the references therein. See [11] (Zhou and Pepe, 1995), [12] (Zhou and Wang, 2000), [13] (Liu, Zhou and Cai, 2009), [14] (Fan and Wang, 2009), [15] (Liu, Wu and Zhou, 2010) censored survival models with auxiliary covariates.

However, due to the direct physical interpretation of the AFT models, and the fact that AFT models are robust to model misspecification in the sense that ignoring a covariate will not lead to much bias in estimating the remaining regression coefficients, see [7] (Cox and Oakes, 1984), the biasing effect of covariate measurement error on AFT models deserves further investigation. A recent work on the subject of measurement error in AFT models was done by [16] (He et al., 2007), using a simulation and extrapolation approach. [17] (Yu and Nan 2010) studied the regression calibration approach within the semiparametric framework, assuming a known parametric relationship between the accurately measured covariates and their auxiliaries, up to a few unknown nuisance parameters. [18] (Granville and Fan, 2012) studied the parametric AFT models with auxiliary covariates based on maximum likelihood method.

In this paper, we study the Buckley-James estimator [19] (Buckley and James, 1979) of AFT models with auxiliary covariates. The Buckley-James estimator was shown by [20] (Jin et al., 2006) to be consistent and asymptotically normal when using a consistent estimator as the initial value, due to its asymptotic linearity. Some other insights about the consistency and asymptotic theory of this estimator has been investigated by [21] (James and Smith, 1984) and [22] (Lai and Ying,1991), among others. We propose a local polynomial approximation method to handle the missing or mismeasured covariates, through the estimation of the conditional expectation of the unobservable estimating functions. This approach makes neither distributional assumptions about the model error term 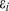, beyond it having mean zero and a finite variance, nor parametric assumptions on the relationship between the correctly measured covariates and their auxiliary variables. The proposed approach will be introduced through a kernel smoothing method, a special case of the local polynomial approximation, see [14] (Fan and Wang, 2009), mainly due to the ease of presentation. See [23] (Nadaraya, 1964), [24] (Watson, 1964), and [25] (Wand and Jones, 1995) for details of kernel smoothing. Intensive simulation studies were conducted to investigate the small sample performance of our proposed method. The results show a remarkable efficiency gain over the method which ignores the auxiliary information.

The remainder of this paper is organized as follows. In the second Section, we introduce Buckley-James estimator for the accelerated failure time model and present our estimation method. Then we investigate the asymptotic properties of our proposed estimator. The Section thereafter contains the results and discussion of our numerical studies, including simulations and the PBC data illustration. In the last Section, we put forth some concluding remarks. The proofs for Theorems were deferred to the appendix.

## Inference Methods of Accelerated Failure Time Model

Let 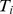 and 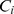, 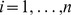 be the failure and censoring times for the *i*th subject in a large study cohort. Due to the censoring, we observe 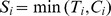 as well as a failure indicator 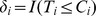. Let 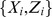 denote the covariate vector where 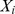 is the component which is only observed in the validation set and 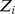 is the component that is available for the full study cohort. Let 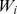 be the auxiliary covariate to 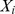. Hence the data consists of the validation sample 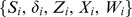, and the nonvalidation sample 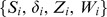. In this paper we assume that 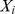 is scalar, mainly because of the simplicity of the presentation, and 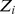 may be either a scalar or a vector. In practice, 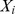 could also be closely correlated with 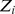. A special case is the classical measurement error model 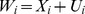, where 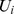 is the error encountered when measuring 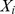. It is assumed that the 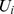
's are independent and identically distributed random normal variables, 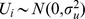. Of the 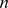 observations, the validation sample contains 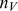 observations, and the non-validation sample contains 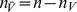 observations.

The accelerated failure time model based solely on the validation sample, can be expressed as
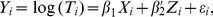
(1)where 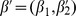 is a vector of unknown regression coefficients and the 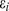
's are independent and identically distributed with an unspecified distribution 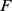 which has mean zero and finite variance. Equation (1) assumes automatically that 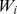 provides no additional information about the failure time, given 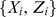.

Without making any assumption to the distribution of 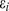, the Buckley-James least squares procedure (Buckley and James, 1979) estimates the regression parameters through the minimization of
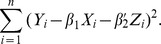



The least squares estimates of 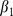 and 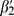 are such that
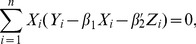
(2)and
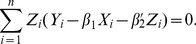
(3)


In order to deal with censoring, let 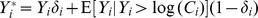. Then 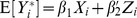, so 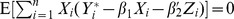, and 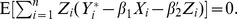


The estimators 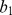 and 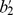 of 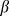 then satisfy
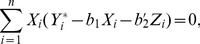
(4)and
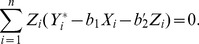
(5)


However, the distribution of 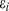 is unknown. The distribution of 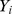, and consequently, 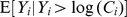 are both unknown. The censored observations are hence replaced by
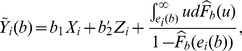
where 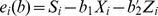, 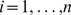 are the residuals, and
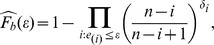
is the Kaplan-Meier Product Limit estimator of the distribution function of the residuals, 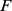. 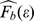 is a discrete function which will not tend to 1 as 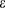 increases if the largest residual is censored. Therefore, following the convention of Buckley and James, the largest residual is redefined as uncensored for all calculations, if necessary.

Let 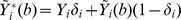. The estimator for 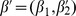 is the solution of the following equation,
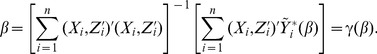
(6)


It should be noted that 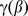 depends on 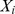, which is available only for the validation sample. For the non-validation sample we can substitute the estimates of their conditional expectations given the auxiliary and other available covariates. The local polynomial approximation approach can be applied for this purpose, see [14] (Fan and Wang, 2009). For the simplicity of the presentation, we use the kernel smoothing method to estimate the conditional expectation of the unobserved covariates given the auxiliary information.

Note that this simplification does not necessarily lead to efficiency loss. Since the direct estimation of the conditional expectation of the estimating function depends also on the Kaplan-Meier estimation of the survival function of the regression residuals, it could also introduce additional instability into the inference, as compared with imputing the estimated conditional expectation of the mismeasured covariate. Our simulation also revealed this observation (results not included).

The conditional expectation of the mis-measured covariate, denoted by 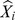, can be estimated as
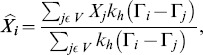
(7)where 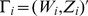, 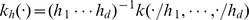 is the kernel function and 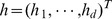 is the chosen vector of bandwidth. Using these 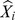
's in place of the 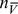 missing 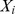
's, we may solve (6) for 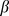 using a numerical method, like Broyden's method. Note that, Broyden's method requires two initial values, while the method of [21] (James and Smith, 1984) only requires a single initial value. In this function, 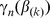 is the value of (6) when calculated using 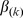. A very natural selection of the initial value of 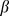 is the least squares regression estimator calculated from the validation sample.

The standard deviation of these estimators is estimated using bootstrapping. For 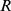 replicates, a simple random sample with replacement of the full sample size is taken from the observed data and the above estimation method for 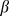 is repeated on each replicate. A sample standard deviation is then calculated to estimate the true standard deviation of the 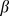 estimators.

**Remark 1** *In order to retain the same quality of information among the replicated estimations, an alternative method of resampling was attempted to keep the proportion of censored observations constant in each replication. We defined 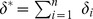 to be the total number of observations with uncensored failure times. For 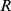 replicates, a simple random sample with replacement of size 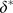 was taken from these uncensored observations, and a simple random sample with replacement of size 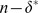 was taken from the remaining censored observations. However this alternative method was found to underestimate the true standard deviations and resulted in coverage probabilities that were lower than the nominal level. The reason of this outcome is mostly due to the fact that the independence of the censoring mechanism was broken by the sampling method.*

### Defining a Solution

In order to solve the estimating equations for the regression parameters, we use the iterative scheme of 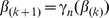. However, as noted by [19] (Buckley and James, 1979) and [21] (James and Smith, 1984), these iterations need not converge. The 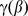 function is discontinuous and piecewise linear in 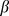 so an exact solution may not exist. When this is the case, the iterations can oscillate between two values of 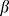. We define a possible alternate solution which is closest to satisfying 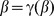, or 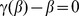. If 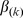 is oscillating between two points due to the lack of an exact solution, we define the alternate solution as 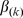 that minimizes the modulus of this difference,
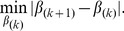
(8)


When the iterations do not converge, a cut-off point has to be determined to stop the iterations. It is advised to select a number of iterations that is slightly greater than the amount required for the convergence when a solution typically does exist. For most of our simulations, we have set this point at 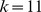. In many cases, our simulations converge in three or four steps. So at 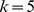 or 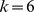, 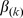 breaks the loop when checked against 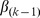 for convergence, implying the solution being reached at iteration 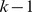. If the iteration does not converge, the first ten values are checked and whichever value, after, say 5 steps of iteration, satisfies equation (8) is selected as a solution.

When dealing with real data, it is advised to choose an arbitrarily large number for the cut-off point to find the best possible solution.

## Asymptotics

In this section, we investigate the asymptotic properties of our proposed estimator. For that sake, we rewrite the estimating function and the Kaplan-Meier estimator of the residual survival function in the counting process frame work. Define a function 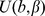 by
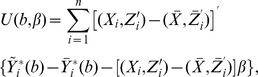
where 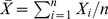, 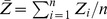 and 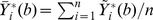. The estimating equation (6) can be rewritten as
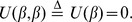



The Buckley-James estimator solves the above equation.

When some of the covariates are accurately recorded only for the validation sample, but with relevant auxiliary information available for the whole study cohort, the estimating functions involved those mis-measured covariates belonging to the nonvalidation sample. We propose to estimate those terms by using the local polynomial approximation approach. Let 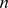 denote the size of whole study cohort, 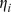, for 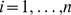 be the validation indicator. Define
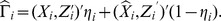



The derived estimating equation is then
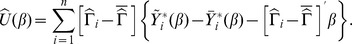



Our proposed estimator of the regression parameter, accommodating the auxiliary information, 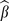 solves this derived estimating equation.

For a vector 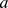, define 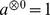, 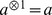, 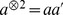 and 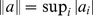. Let 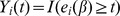, for 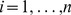 and 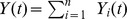. Let 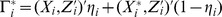, where 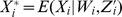 for 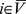.

Let 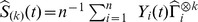, 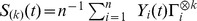 and
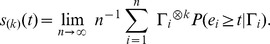



Denote further 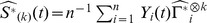, 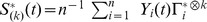 and
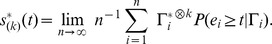



Without loss of generality, let 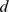 be the dimension of 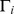 in the definition of the local polynomial approximation. Suppose further that 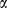 is the order of the kernel function 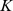, i.e.
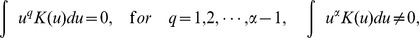
and 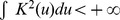. The bandwidth conditions are given below.

[**BC**] As 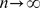, 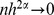, 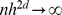.

The following assumptions, beyond the bandwidth conditions, are necessary for the asymptotic properties of the proposed method.

C.0 The hazard rate function 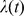 of 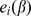 is such that 
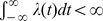
.

C.1 There exists a constant 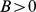, such that 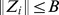, 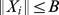 and 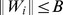 for all 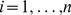.

C.2 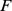 has a twice-continuously differentiable density 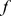 such that
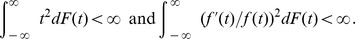



C.3 The solution to 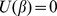 is unique and is an interior point of 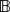, where 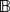 is a compact subset of 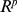.

C.4 There exists a function 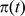 such that, as 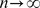,
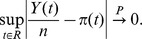



**Remark 2** *The assumption C.3 is proposed just to simplify the proof of the asymptotics of the Buckley-James estimator. This could be violated due to the instability of the Kaplan-Meier estimator of the survival function when getting into the distribution tail. When this violation happens, the tail modification by [22] (Lai and Ying, 1991) should be applied and the method of [26] (Jin, et al., 2006) of selecting a consistent and asymptotically normal estimator as the initial value can be adopted.*

**Theorem 1** *Under conditions C.0-C.4 and the bandwidth conditions [BC], 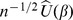 converges in distribution to a zero-mean normal random vector with covariance matrix 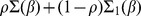, where* 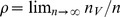,
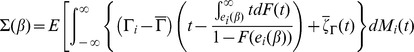


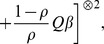

*where* 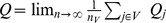,
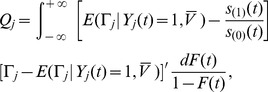

*and* 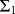 *is defined as*

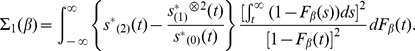



**Theorem 2** *Under assumptions C.0-C.4 and the bandwidth conditions [BC], 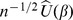 is asymptotically linear within the 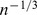 neighborhood of 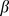, with probability 1, in the sense that*

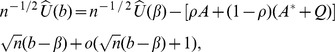

*if* 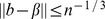, *where the matrix* 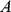 *is defined as*

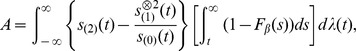

*and 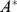 as*

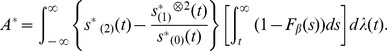



**Corollary 1** *Under assumptions C.0-C.4, the bandwidth conditions [BC] and the assumption that 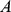 is nonsingular, the solution 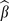 to 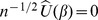 converges in probability to* 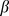.

**Theorem 3** *Under assumptions C.0-C.4 and the bandwidth conditions [BC], 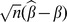 converges in distribution to a zero mean normal random vector with covariance matrix*

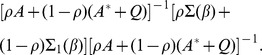



### Proof of Theorem 1

Let 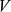 be the set of the indices of the validation sample, 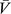 that of the non-validation sample and 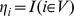, 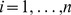 be the validation indicator.

Let 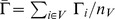, 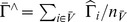 and 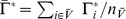. Let
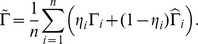



Define
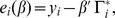
and
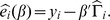



Then
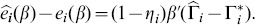



Let 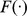 and 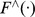 be the distribution functions of 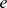 and 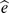, 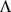 and 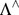 be their cumulative hazard functions. Then
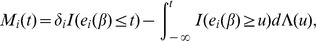
and
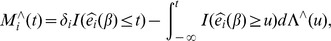
are martingales with respect to complete 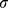-field generated by
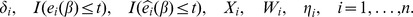



Further,
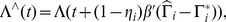
and
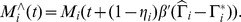



Let 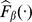 and 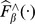 be the (nominal) Kaplan-Meier estimators of 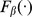 and 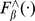. The estimated Buckley-James estimating function can be rewritten as
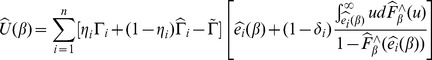


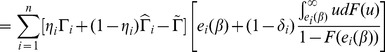


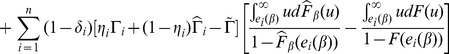


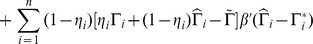


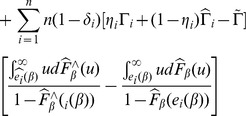


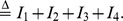



By the martingale representation of Kaplan-Meier estimator of the survival function see [25] (Fleming and Harrington, 1991), also see [20] (Jin, et al., 2006), the bandwidth condition [BC] and the continuity of 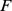 (assumption C.2), the first two terms in the above equation can be rewritten as
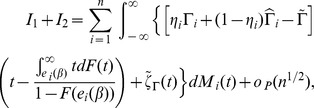
where
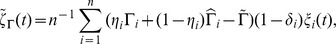
and
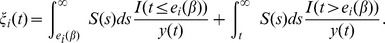



The Kaplan-Meier estimators of the survival functions of 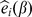 and 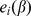 lead to
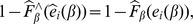
and
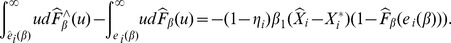



Hence
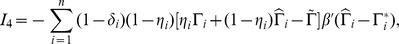
and
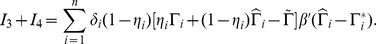



This term can be further rewritten as
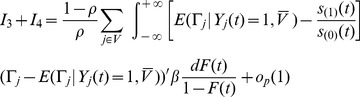


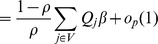



We have
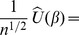


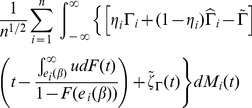


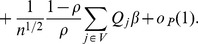



By assumptions C.0, C.2 and Lenglart's inequality, we have
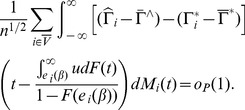



Hence the estimating function can be rewritten as
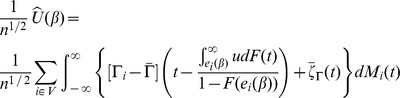


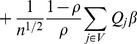


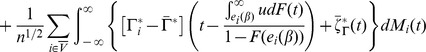


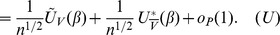



Note that 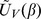 is a sum of 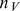 i.i.d. terms hence central limit theorem applies. By conditions C.0 through C.4 and the martingale central limit theorem, 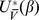 converges in distribution to a normal random vector. Further, by independence of 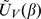 and 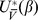, we have
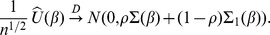



### Proof of Theorem 2

From the equation (U) in the proof of Theorem 3.2, and by Theorem 4.1 of Lai and Ying (1991), we have, for 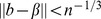,
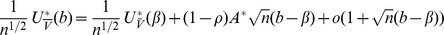
with probability 1. The term 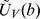 consists of two parts, we have
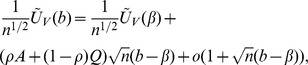
with probability 1. Hence
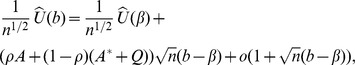
with probability 1.

Corollary 1 and Theorem 3 are direct conclusions of Theorems 1 and 2.

## Results of Numerical Studies

### Simulation Studies

In this section we examine the small sample performance of our proposed estimator. Let 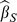 denote our proposed estimator of the regression coefficients. Its small sample performance is compared with three alternative estimators: the validation estimator (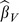) which is based solely on the validation sample; the naive estimator (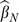), which ignores the measurement error by assuming that the unobserved 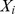
's are equal to the observed 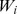
's; and the complete case estimator (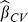), when we assume that 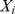 are observed for the whole study cohort.

The data for the simulations was generated in the following way. The 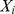 and 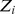 are generated from a uniform distribution, 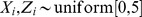. For each 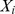, the auxiliary covariate is defined as 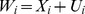, where 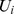 is generated from a normal distribution with mean zero and standard deviation 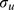. The value of 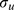 determines the magnitude of the measurement error. The failure times were then defined as 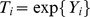 where 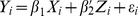. The 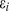
's were taken to be independent and identically distributed from either a standard normal, standard extreme value, or logistic distribution, respectively.

Various other parameters are controlled over all simulations. Each run calculates 1000 replicates in the bootstrapping to give consistent estimators of the standard deviations. The parameters were chosen as 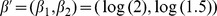. Within a simulation, the censoring times are randomly generated from a uniform distribution with lower limit 0 and an appropriate upper limit to ensure an approximate 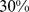 or 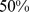 censor rate. The 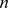 and 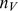 values are chosen to be either 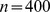 and 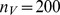, having half of the data in the validation set, or 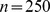 and 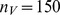, with the validation set containing 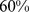 of the data. Finally, two values of 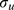 are selected, 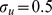, and 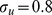. For the kernel smoothing used to calculate 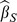 the Gaussian kernel function is selected, which has an order of 2,
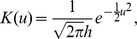
where 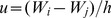. We choose bandwidth 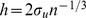 as used by [12] (Zhou and Wang, 2000).

The standard error (SE), standard deviation (SD), and coverage probability (CP) are calculated for each set of simulations. The SE values are the sample standard deviations of the 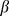 estimates, the SD values are the mean standard deviations generated from the bootstrapping in each simulation, and CP is equal to the percentage of simulations that had the true 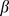 value within a 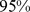 confidence interval around its estimate when using the result of the bootstrapping for the standard deviation. The results are presented in Tables 1, 2, and 3.

**Table 1:** Results after 500 simulations for 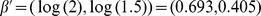 using a standard normal error term.

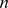	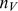	Censor Rate	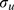	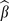	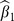	SD 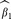	SE 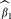	CP 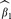	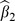	SD 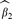	SE 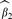	CP 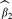
400	200	0.3	0.5	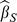	0.693	0.030	0.031	0.948	0.405	0.029	0.029	0.950
				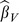	0.692	0.041	0.044	0.924	0.407	0.040	0.041	0.938
				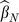	0.673	0.030	0.030	0.882	0.423	0.029	0.029	0.924
				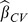	0.693	0.029	0.030	0.954	0.406	0.028	0.028	0.950
400	200	0.5	0.5	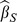	0.692	0.035	0.036	0.940	0.405	0.032	0.033	0.934
				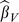	0.692	0.049	0.048	0.942	0.406	0.044	0.045	0.948
				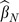	0.676	0.035	0.035	0.894	0.424	0.032	0.033	0.902
				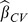	0.691	0.034	0.035	0.936	0.406	0.031	0.032	0.936
400	200	0.3	0.8	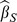	0.695	0.031	0.032	0.948	0.404	0.030	0.031	0.928
				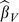	0.691	0.041	0.040	0.964	0.408	0.040	0.040	0.948
				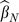	0.645	0.031	0.031	0.650	0.449	0.030	0.030	0.676
				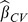	0.693	0.029	0.030	0.944	0.405	0.028	0.029	0.938
400	200	0.5	0.8	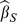	0.695	0.036	0.037	0.956	0.400	0.033	0.035	0.932
				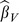	0.694	0.049	0.053	0.938	0.403	0.044	0.046	0.942
				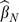	0.655	0.036	0.036	0.782	0.451	0.033	0.035	0.728
				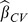	0.694	0.034	0.034	0.958	0.403	0.031	0.033	0.944
250	150	0.3	0.5	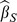	0.690	0.038	0.039	0.938	0.408	0.036	0.038	0.938
				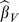	0.688	0.048	0.049	0.938	0.408	0.046	0.045	0.940
				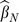	0.674	0.038	0.039	0.914	0.422	0.036	0.037	0.910
				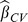	0.689	0.037	0.038	0.942	0.407	0.035	0.037	0.938
250	150	0.5	0.5	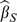	0.690	0.044	0.044	0.936	0.402	0.040	0.040	0.944
				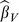	0.690	0.056	0.058	0.940	0.404	0.050	0.051	0.952
				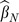	0.677	0.044	0.044	0.920	0.418	0.040	0.040	0.944
				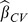	0.690	0.044	0.044	0.938	0.403	0.039	0.039	0.950

**Table 2:** Results after 500 simulations for 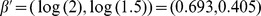 using an extreme value error term.

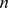	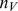	Censor Rate	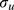	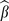	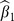	SD 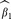	SE 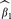	CP 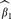	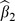	SD 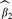	SE 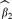	CP 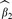
400	200	0.3	0.5	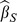	0.696	0.058	0.056	0.962	0.411	0.054	0.056	0.948
				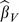	0.694	0.080	0.078	0.946	0.413	0.076	0.080	0.938
				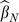	0.657	0.055	0.053	0.892	0.411	0.054	0.056	0.946
				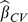	0.696	0.056	0.054	0.954	0.411	0.054	0.056	0.944
250	150	0.3	0.5	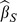	0.694	0.073	0.076	0.940	0.401	0.068	0.067	0.960
				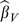	0.695	0.092	0.095	0.926	0.399	0.086	0.090	0.940
				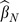	0.663	0.070	0.072	0.920	0.401	0.068	0.067	0.958
				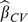	0.694	0.071	0.072	0.948	0.402	0.067	0.066	0.960

**Table 3:** Results after 500 simulations for 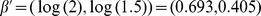 using a logistic error term.

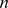	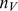	Censor Rate	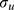	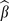	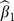	SD 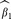	SE 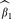	CP 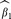	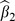	SD 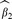	SE 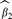	CP 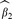
400	200	0.3	0.5	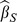	0.689	0.069	0.071	0.934	0.405	0.066	0.067	0.952
				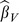	0.693	0.095	0.099	0.940	0.403	0.094	0.092	0.956
				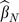	0.651	0.065	0.068	0.888	0.406	0.066	0.067	0.948
				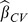	0.688	0.067	0.070	0.940	0.405	0.066	0.067	0.956
400	200	0.3	0.8	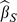	0.701	0.072	0.074	0.936	0.403	0.067	0.071	0.926
				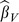	0.701	0.096	0.096	0.950	0.401	0.093	0.096	0.942
				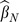	0.608	0.064	0.064	0.724	0.403	0.067	0.071	0.932
				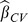	0.697	0.067	0.070	0.936	0.403	0.066	0.070	0.926

From Tables 1, 2, and 3, we make the following observations:

- Estimators 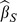 and 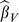 are performing very well for each of the three error distributions.
- Naive estimator 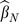 is biased when the measurement error variance 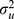 is large.
- The 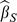 estimator is more efficient than 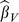, having standard errors comparable to that of 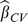.
- The proposed method removes the estimation bias in 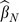, both for the regression coefficient of the error-prone covariate and that of the accurately measured covariates.
- The bootstrapping procedure results in good estimates of the standard error for all observed cases over the four estimators and three error distributions.
- The coverage probabilities for the 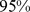 confidence intervals are very close to their nominal level, except for 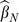 when 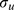 is large, where the estimate is severely biased.
- The model experiences the least variation when the error term follows the standard normal distribution, with standard errors that are approximately half the size as when the error term follows the chosen extreme value or logistic distributions.
- The efficiency gain is ignorable when 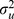 is small, such as 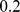 or smaller (simulation results not reported).

### Application to PBC Data

To illustrate how to use the smoothing method in practice, we analyze the data from the Mayo Clinic trial in primary biliary cirrhosis (PBC) of the liver. PBC is a chronic liver disease that inflames and slowly destroys the bile ducts in the liver, impairing its ability to function properly. It is believed to be a type of autoimmune disorder where the immune system attacks the bile ducts. PBC occurs primarily in women, with approximately 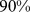 of patients being women, most often between the ages of 40 and 60. There is currently no known cure for the disease; the only known way to remove PBC is through a liver transplant, see [27], [28].

In the Mayo Clinic trial, 418 patients were eligible. Of these patients, mostly complete data was obtained from the first 312 patients. The remaining 106 patients were not part of the actual clinical trial but had some basic measurements taken and were followed for survival. The variables we used in our regression on the logarithm of time were *age*, patient's age (in years); *albumin*, serum albumin (in mg/dl); *ast*, aspartate aminotransferase (in U/ml), once referred to as SGOT; *bili*, serum bilirunbin (in mg/dl); *copper*, urine copper (ug/day); *edema*, equal to 0 if no edema, 0.5 if untreated or successfully treated, or 1 if there exists edema despite diuretic therapy; *protime*, standardized blood clotting time. Of these, two cases were examined using either ast or copper for our 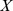 covariate to be smoothed due to incomplete data, while the others are mostly complete and thus are included in 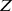.

Edema was split into two categorical variables, *edema05* and *edema1*, defined as
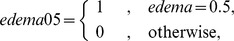
and
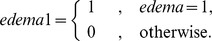



We also took the log transformation of *albumin*, *ast*, *bili*, *copper*, and *protime*, in the interest of making their marginal distributions closer to normal. For the smoothing of the unobserved 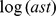 and 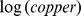 values, 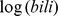 was chosen as the auxiliary covariate for both due to its high correlation (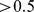) with both variables. The bandwidth 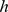 was calculated using the sample standard deviation of 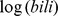, resulting in 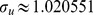 and 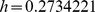 using the same formula as in the numerical simulations, 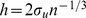. Any observations missing a value for either of the 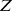 covariates were removed, leaving both cases with 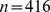 while 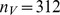 for the model using 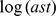 and 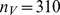 for the model using 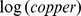.

Examining Tables 4 and 5, we see that both 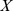 variables had their estimated standard deviations increase by a small amount due to the error added into the model from smoothing for a missing covariate instead of a mismeasured one. If 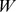 was of the form 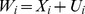 like in the simulations, it could have resulted in a higher correlation between the auxiliary variable, 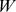, and the 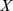 variable, depending on the magnitude of the measurement error. Despite the small increase in standard deviation the 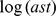 term becomes significant at the 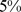 confidence level after smoothing, and while 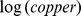 was already significant, the p-value did decrease. For the 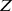 variables, we see that they all have a smaller or approximately equal standard deviation after smoothing, which is expected when using the full sample size without needing smoothing for those variables, except for 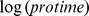 and the intercept term which increased.

**Table 4:** AFT model analysis of PBC data, smoothing for 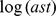.

Covariate	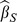	SD	P-Value	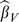	SD	P-Value
Intercept	15.5304	2.5729	1.5792e-09	16.1642	2.3047	2.3239e-12
log(ast)	−0.3783	0.1926	4.9482e-02	−0.3364	0.1805	6.2311e-02
age	−0.0278	0.0058	1.8556e-06	−0.0249	0.0061	3.9895e-05
log(albumin)	1.4729	0.5551	7.9733e-03	1.3926	0.5883	1.7931e-02
log(bili)	−0.4800	0.0781	7.7648e-10	−0.4510	0.0781	7.7448e-09
edema05	−0.4387	0.2124	3.8858e-02	−0.3006	0.2221	1.7593e-01
edema1	−0.9190	0.2968	1.9610e-03	−0.9178	0.3063	2.7279e-03
log(protime)	−2.4323	0.8712	5.2415e-03	−2.8227	0.7813	3.0267e-04

**Table 5:** AFT model analysis of PBC data, smoothing for 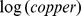.

Covariate	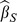	SD	P-Value	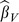	SD	P-Value
Intercept	14.6413	2.1482	9.3809e-12	15.1929	1.8216	0.0000e+00
log(copper)	−0.3299	0.0883	1.8663e-04	−0.3105	0.0873	3.7675e-04
age	−0.0250	0.0061	3.9593e-05	−0.0217	0.0061	3.4084e-04
log(albumin)	1.4324	0.5499	9.1876e-03	1.2576	0.5783	2.9666e-02
log(bili)	−0.4218	0.0717	3.9422e-09	−0.4018	0.0739	5.3323e-08
edema05	−0.4285	0.2160	4.7314e-02	−0.3097	0.2226	1.6422e-01
edema1	−0.9021	0.3041	3.0152e-03	−0.9411	0.3113	2.5003e-03
log(protime)	−2.2738	0.8185	5.4687e-03	−2.5324	0.7294	5.1651e-04

## Discussion

In this paper we proposed the use of the Buckley-James estimator as a nonparametric method of estimating the regression parameters of an accelerated failure time model with auxiliary covariates. Kernel smoothing was applied using the auxiliary covariates to estimate missing or mismeasured covariates. The Buckley-James method is then applied to the whole study cohort for the inference of the covariates effect. The standard deviations of the estimates of the regression coefficients are estimated through bootstrapping. The proposed estimator is consistent and asymptotically normal.

This method was most effective in the case of mis-measured data due to the naturally high correlation between the corresponding 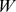 and 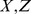 variables which resulted in the estimator involving the smoothing, 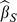, being more efficient than the validation estimator, 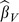, as shown in the numerical simulations. The method should also perform well for the missing variable case given a sufficiently strong correlation. The method was applied to the PBC data as an illustration.

The smoothing model is set up in a general format. In applications, we should only choose those variables which are highly related to the mismeasured one. By doing so we can avoid the situations such as the auxiliary covariates only occupy a narrow region, which could cause instability in the local smoothing, hence the whole model.

Caution should also be taken when the proposed method is applied to a data with extremely small validation sample. A classic measurement error model might be a better option, where one can estimate the measurement error variance using the validation sample.

## References

[1] Prentice RL (1982) Covariate measurement errors and parameter estimation in failure time regression model. Biometrika 69 pp 331–342.

[2] Rubin DB (1976) Inference and missing data. Biometrika 63 pp 581–592.

[3] Fuller WA (1987) Measurement Error Models, Wiley, NewYork.

[4] Carrol RJ, Rupert D, Stefanski LA (1995) Measurement Error in Nonlinear Models, Chapman and Hall, London.

[5] Wang N, Lin X, Gutierrez RG, Carrol RJ (1998) Bias analysis and SIMEX approach in generalized linear mixed measurement error models. J. Am. Statist. Assoc 93 pp 249–261.

[6] Cox DR (1972) Regression models and life-tables (with discussion). J. R. Stat. Soc. Ser. B 34 pp 187–220.

[7] Cox DR, Oakes D (1984) Analysis of Survival Data, Chapman and Hall, London.

[8] Kalbfleisch JD, Prentice RL (2002) The Statistical Analysis of Failure Time Data, Second Edition, John Wiley & Sons, Inc., Hoboken, NJ, USA.

[9] Hu P, Tsiatis AA, Davidian M (1998) Estimating the parameters in the Cox model when covariate variables are measured with error. Biometrics 54 pp 1407–1419. 9883541

[10] Hu C, Lin D (2002) Cox regression with covariate measurement error. Scandinavian Journal of Statistics 29 pp 637–655.

[11] Zhou H, Pepe MS (1995) Auxiliary covariate data in failure time regression analysis. Biometrika 82 pp 139–149.

[12] Zhou H, Wang CY (2000) Failure Time Regression with Continuous Covariates Measured with Error. Journal of the Royal Statistical Society. Series B (Statistical Methodology) Vol. 62 No. 4, pp 657–665.

[13] Liu Y, Zhou H, Cai J (2009) Estimated pseudopartial-likelihood method for correlated failure time data with auxiliary covariates. Biometrics 65 pp 1184–1193. 19432779 10.1111/j.1541-0420.2009.01198.x PMC2819485

[14] Fan Z, Wang X (2009) Marginal hazards model for multivariate failure time data with auxiliary covariates. Journal of Nonparametric Statistics 21 7, pp 771–786.

[15] Liu Y, Wu Y, Zhou H (2010) Multivariate failure time regression with a continuous auxiliary covariate. Journal of Multivariate Analysis 101 pp 679–691. 21966052 10.1016/j.jmva.2009.09.008 PMC3182102

[16] He W, Yi GC, Xiong J (2007) Accelerated failure time models with covariates subject to measurement error, Statist. Med. 26 pp 4817–4832. 10.1002/sim.2892 17436310

[17] Yu M, Nan B (2010) Regression Calibration in Semiparametric Accelerated Failure Time Models. Biometrics 66 pp 405–414. 19645700 10.1111/j.1541-0420.2009.01295.x

[18] Granville K, Fan Z (2012) Accelarated Failure Time Models with Auxiliary covariates. J Biom Biostat 3: 152 doi:10.4172/2155-6180.1000152

[19] Buckley J, James I (1979) Linear regression with censored data. Biometrika 66 (3) pp 429–436.

[20] Jin Z, Lin DY, Ying Z (2006) On least-squares regression with censored data. Biometrika 93 (1) pp 147–161.

[21] James IR, Smith PJ (1984) Consistency results for linear regression with censored data. The Annals of Statistics Vol. 12 No. 2, pp 590–600.

[22] Lai TL, Ying Z (1991) Large sample theory of a modified Buckley-James estimator for regression analysis with censored data. The Annals of Statistics Vol. 19 No. 3, pp 1370–1402.

[23] Nadaraya TA (1964) On estimating regression. Theory Probab. Applic 10 pp 186–190.

[24] Watson GS (1964) Smooth Regression Analysis. Sankhyā: The Indian Journal of Statistics, Ser. A 26 pp 359–372.

[25] Wand M, Jones M (1995) Kernel Smoothing, Chapman and Hall, London.

[26] Fleming TR, Harrington DP (1991) Counting Processes and Survival Analysis, Jone Wiley & Sons, Inc. New York.

[27] National Institutes of Health (2011) “Primary Biliary Cirrhosis.” National Digestive Diseases Information Clearinghouse (NDDIC). December 2008. National Institute of Diabetes and Digestive and Kidney Diseases, National Institutes of Health. 29 July 2011. Available: http://digestive.niddk.nih.gov/ddiseases/pubs/primarybiliarycirrhosis/. Accessed 2014 Jul 25.

[28] “Primary Biliary Cirrhosis (PBC).” American Liver Foundation. 22 March 2011. 29 July 2011. Available: http://www.liverfoundation.org/abouttheliver/info/pbc/. Accessed 2014 Jul 25.

